# Pathologic Cellular Events in Smoking-Related Pancreatitis

**DOI:** 10.3390/cancers7020723

**Published:** 2015-04-29

**Authors:** Edwin Thrower

**Affiliations:** 1 Department of Internal Medicine, Section of Digestive Diseases, Yale University School of Medicine, New Haven, CT 06520, USA; E-Mail: Edwin.thrower@yale.edu; Tel.: +1-203-932-5711 (ext. 3284); Fax: +1-203-937-3852; 2 Veterans Affairs Connecticut Healthcare, West Haven, CT 06516, USA

**Keywords:** pancreatitis, smoking, nicotine, 4-(methylnitrosamino)-1-(3-pyridyl)-1-butanone (NNK), nicotinic acetylcholine receptors (nAChRs), inflammation, bioactivation, β-adrenergic receptors

## Abstract

Pancreatitis, a debilitating inflammatory disorder, results from pancreatic injury. Alcohol abuse is the foremost cause, although cigarette smoking has recently surfaced as a distinct risk factor. The mechanisms by which cigarette smoke and its toxins initiate pathological cellular events leading to pancreatitis, have not been clearly defined. Although cigarette smoke is composed of more than 4000 compounds, it is mainly nicotine and the tobacco-specific nitrosamine 4-(methylnitrosamino)-1-(3-pyridyl)-1-butanone (NNK), which have been extensively studied with respect to pancreatic diseases. This review summarizes these research findings and highlights cellular pathways which may be of relevance in initiation and progression of smoking-related pancreatitis.

## 1. Introduction

Pancreatitis is an inflammatory condition which arises following injury to the pancreas. An early pivotal event in initiation of acute pancreatitis (AP) is premature activation and retention of digestive pro-enzymes (zymogens) in pancreatic acinar cells, leading to autodigestion of the pancreas [1]. Inflammation, edema, ischemia, and cell death subsequently follow [2]. Typically, AP is self-limiting, with a complete recovery after the acute event. However, recurrent bouts of AP can set in motion inflammatory events that in turn lead to stellate cell activation and increased fibrosis, resulting in chronic pancreatitis (CP) [3]. Chronic inflammation associated with CP facilitates progression to cancer, as seen by formation of precancerous lesions and subsequent advancement to pancreatic ductal adenocarcinoma (PDAC). Furthermore, patients with CP are found to be at elevated risk for developing pancreatic cancer [4].

Usual etiologies for pancreatitis include alcohol abuse and gallstones although several recent clinical studies have identified cigarette smoking as an independent and dose-dependent cause for both AP and CP [5,6,7,8,9,10,11,12,13,14,15,16,17]. The pathological cellular mechanisms accounting for these risks, however, remain largely unknown. Some scientific studies have investigated effects of cigarette smoke inhalation in rats; animals developed pancreatic injury and elevated levels of pancreatic zymogens, although the cellular damage was less than that observed in human CP [18,19]. Given that cigarette smoke and tobacco contain a large number of toxic products, other studies have focused specifically on nicotine and its metabolites, which are among the most abundant of these toxins, and have been heavily implicated in disease. In subsequent sections, we will further explore recent research looking at effects and mechanisms mediated by nicotine and the tobacco-specific nitrosamine 4-(methylnitrosamino)-1-(3-pyridyl)-1-butanone (NNK), and their potential role in development of pancreatitis.

## 2. Induction of Pancreatitis Responses by Cigarette Toxins

### 2.1. Nicotine

Nicotine is considered to be the most likely candidate in cigarettes and tobacco products for initiating smoking-related illnesses. Studies have demonstrated that nicotine plays a substantial part in induction of pancreatic pathophysiology [20,21,22,23,24,25]. Elevated levels of nicotine metabolites have been measured in human pancreatic juice collected from smokers. Cotinine, a primary metabolite, was present at levels around 130 ng/mL whereas NNK ranged from 1.37 ng/mL to 600 ng/mL (0.7 µM and 6.6 nM–3 µM respectively) [26]. Animal studies have also validated that the pancreas receives significant exposure to nicotine: Inhalation of ^3^H-nicotine by rats resulted in an accumulation in the pancreas and intestine [21,27]. When rats were exposed to graded doses of nicotine for a period of 3–16 weeks through aerosol or feeding (intragastric or *ad-libitum*) pathological changes occurred in exocrine pancreatic tissue. Cytoplasmic swelling, vacuolization, formation of pyknotic nuclei and karyorrhexis were observed in exocrine pancreatic cells. Furthermore, isolated acinar cells harvested from untreated animals were subsequently exposed to nicotine and incurred similar pathological damage. These changes reproduce those viewed in acute or experimental pancreatitis [22,23,28,29,30,31].

Nicotine, when given at pharmacological concentrations to animals *in vivo*, reduced exocrine function via suppression of pancreatic amylase release [25,30,32,33]. At low concentrations (0.1 nM) the hormone cholecystokinin (CCK) stimulates normal physiological responses in isolated rat pancreatic acini, including amylase secretion. Nicotine exposure, at low concentrations (100 µM) augments this CCK-induced amylase secretion, however at higher concentrations (up to 1 mM) it reduces it [34,35]. This reduction in secretion by high nicotine levels is similar to that seen in experimental models of pancreatitis, whereby stimulation of acinar cells with *hyperstimulatory* concentrations of CCK (10–100 nM) induces early stages of acute pancreatitis [1]. Premature activation of digestive enzymes and reduced secretion results in retention of active digestive enzymes within the acinar cell, and contributes to the degradation of the pancreas during the course of the disease. Nicotine-induced secretory events in isolated rat acini are abrogated following treatment with mecamylamine or ω-conotoxin, nicotinic receptor antagonists, and the calcium channel antagonist, 2-Aminoethoxydiphenyl borate (2-APB) [35]. This evidence suggests that nicotine might induce cellular responses through a nicotinic acetylcholine receptor (nAChR) and that calcium functions as a downstream effector. The role of nAChR and calcium signaling in smoking-related pancreatitis will be considered in more detail in Section 3.1.

Circulating levels of the gastrointestinal hormones gastrin and CCK were affected by nicotine exposure in rats [36]. Fluctuations in basal levels of these hormones, as well as serum enzymes such as amylase and lipase, have been connected with morphological variations which occur in pancreatitis [21,34]. Nicotine can also regulate lipid peroxidation and oxidative stress although it is uncertain if these processes participate in pancreatic pathophysiology [34].

Nicotine may alter the proteome of pancreatic cells, increasing expression of proteins that may be involved in pancreatitis and other pancreatic diseases. The effects of nicotine on the proteomes of two pancreatic duct cell lines—an immortalized normal cell line (HPNE) and a cancer cell line (PanC1)- were investigated using mass spectrometry-based proteomics [37]. Over 5000 proteins were detected per cell line. Of these, more than 900 proteins were differentially expressed upon nicotine treatment, 57 of which were detected in both cell lines. In particular, this study emphasized that amyloid precursor protein (APP), previously observed to have increased expression in pancreatic stellate cells upon nicotine treatment [38] was also up-regulated in both ductal cell lines. Although the role of APP in pancreatic physiology is unclear, its increased expression may be connected to inflammatory or fibrotic responses. These data imply that nicotine may play a significant role in the initiation and progression of pancreatic disease.

### 2.2. NNK

NNK, a tobacco-specific nitrosamine derived from nicotine, is one of the most harmful components of cigarette smoke. Recently, NNK was defined as an initiator of, and sensitizer to, AP. Studies using isolated rat acinar cells and *in vivo* models of pancreatitis [39] found that NNK treatment induced a key event in initiation of pancreatitis: premature activation of digestive zymogens (trypsinogen and chymotrypsinogen). Secondly, the effects of NNK in combination with a frequently-used model of pancreatitis (the “cerulein” model) were explored, to see if NNK pre-treatment could increase pancreatitis responses. Cerulein, an orthologue of the hormone cholecystokinin (CCK), when given at supraphysiologic concentrations (10–100× that required to induce physiological responses), induces typical pancreatitis responses (zymogen activation, histological/morphological changes) in isolated acinar cells or live animals. Pre-exposure to NNK, followed by cerulein stimulation, raised zymogen activation to levels greater than that observed with a single application of either NNK or cerulein. Furthermore, NNK triggered cellular injury in pancreatic tissue (vacuolization, pyknotic nuclei, and edema) analogous to that seen during AP.

The cellular mechanisms through which nicotine and NNK inflict damage upon the pancreas are largely undetermined. Several studies have used a combination of pharmacological and biochemical approaches to identify pathways underlying initiation and progression of pancreatitis and other pancreatic diseases. These findings will be explored in Section 3.

## 3. Cellular Mechanisms Mediated by Cigarette Toxins

### 3.1. Calcium Signaling

Intracellular calcium overload is a critical early stage in the pathogenesis of numerous diseases. In pancreatic acinar cells, alcohol metabolites, bile and other factors initiate a sustained elevation of global calcium, resulting in premature trypsin activation, vacuolization and cell death (necrosis or apoptosis), all of which are necessary for the development of pancreatitis [40]. Stimulation of G-protein coupled receptors on the acinar cell surface generates second messengers such as inositol (1,4,5)-trisphosphate (IP_3_), cyclic ADP ribose (cADPR), and nicotinic acid adenine dinucleotide phosphate (NAADP). These second messengers can activate calcium channels (IP_3_ receptor and Ryanodine receptor) on the surface of the endoplasmic reticulum (ER) calcium store causing a pathological elevation in intracellular calcium. Alternatively calcium can enter through the plasma membrane via so-called “store-operated” calcium channels (SOC), although the mechanism is unclear. Furthermore, failed mitochondrial adenosine triphosphate (ATP) production can lower re-uptake and extrusion of calcium by ATP-dependent calcium pumps in the ER (sarco/endoplasmic reticulum Ca^2+^-activated ATPase) and plasma membrane [40].

Whether nicotine and NNK mediate their effects on the pancreas, particularly the acinar cell, through aberrant calcium dynamics is open to question. A recent study, with isolated rat pancreatic acinar cells, showed that enhanced secretory responses induced by nicotine were suppressed by a calcium-selective antagonist [35]. The antagonist 2-APB, however, blocks both store-operated calcium entry *and* IP_3_-induced calcium release, so this particular study does not clearly reveal *how* nicotine elevates calcium [41]. In this same study, the nAChR blocker, mecamylamine, abrogated nicotine-stimulated responses, implying the additional involvement of nAChR. The α7 isoform of nAChR is also a calcium channel, and it is possible that the elevations in intracellular calcium may occur when nicotine activates it, allowing passage of calcium from the extracellular to intracellular environment [42]. Alternately, it has been shown that choline activation of α7 nAChR promotes a rise in intracellular calcium from local ER stores via G-protein (Gαq) signaling, leading to IP_3_ receptor (IP_3_R) activation at the growth cone of differentiating PC12 cells [43]. Potentially, a similar mechanism in acinar cells may be responsible for pathologically high calcium levels. It should be noted that although mecamylamine blocked nicotine effects in pancreatic acini, the presence of non-neuronal α7 nAchRs was not explored in this particular study. In Section 3.2, further evidence supporting a role for α7 nAchR in smoking-related pancreatitis will be described.

### 3.2. Nicotinic Acetylcholine Receptors

Nicotinic acetylcholine receptors were originally identified within the nervous system, but have subsequently been located in non-neuronal cells [44]. Some cancer cell lines, human keratinocytes, and epithelial cells have α7 nAChR and are sensitive to NNK treatment (EC_50_ for NNK = 0.03 µM). NNK is found in tobacco smoke at concentrations 5000–10,000 times less than nicotine but has a 1000-fold higher affinity for α7 nAChR in comparison. In the organs of smokers, and in the pancreas and lungs of rodents that have been subjected to chronic nicotine/NNK exposure, α7 nAchRs are up-regulated [44,45].

A recent study by Alexandre *et al.* had recorded effects of NNK in induction of pancreatitis responses (described in Section 2.2). They extended this study to determine the cellular target of NNK. Non-neuronal α7 nAChR was selected as a potential candidate and it was primarily established that the receptor was in fact present in rat pancreatic acini by PCR analysis [39]. A functional role was confirmed when isolated acini were pre-treated with mecamylamine, and NNK induced zymogen activation was annulled. These findings were further substantiated when isolated pancreatic acini from α7 nAChR^−/−^ mice failed to respond to NNK exposure and no premature zymogen activation was seen when compared with wild type [46]. Whether additional pancreatitis responses are mediated through the α7 nAChR and related pathways is a subject for future research.

### 3.3. Inflammatory Responses

In addition to direct interactions with acinar cell α7 nAChRs, both NNK and nicotine could potentially regulate immune responses during pancreatitis via α7nAChRs expressed on macrophages. Nicotine has been found to prevent NFκB activation in macrophages, thus obstructing generation of pro-inflammatory cytokines responsible for macrophage stimulation [30,47]. Furthermore, administration of mecamylamine to mice reduced neutrophil and macrophage migration to pancreatic tissue, resulting in more severe experimental pancreatitis [48]. In another study, prophylactic and delayed therapeutic application of nicotine significantly diminished the severity of acute experimental pancreatitis in rats via induction of the cholinergic anti-inflammatory pathway [49]. In a model of severe acute pancreatitis (SAP), induced in mice by retrograde injection of 2% Na-taurocholate into the pancreatic duct, nicotine treatment was found to have a protective effect [50]. Nicotine (50–300 µg/kg) inhibited tissue injury, digestive enzyme production, and pro-inflammatory cytokine generation in a dose-dependent fashion. Additionally, nicotine up-regulated the number and suppressive capacity of CD4^+^ CD25^+^ regulatory T cells (Treg) by causing expression of immunoregulatory molecules and secretion of transforming growth factor β1 (TGF-β1).

The concept of nicotine and NNK being responsible for anti-inflammatory effects and reduction in severity of pancreatitis may seem to conflict with other studies demonstrating a role in disease initiation [20,21,22,23,24,25,39]. However, it is known that prolonged exposure to cigarette smoke causes chronic pancreatic inflammation. Additional studies suggest that NNK might initiate pro-inflammatory pathways in macrophages and other cells through its up-take and metabolism, a process known as “bioactivation” [51]. This process occurs via the cytochrome P450 (CYP450) enzyme family through three major pathways: (a) carbonyl reduction, (b) pyridine *N*-oxidation and (c) α-hydroxylation. Following bioactivation, NNK metabolites can mediate a number of pathological cellular pathways. In U937 human macrophages, for example, NNK metabolites activated NFκB, inducing TNF-α release, while impeding synthesis of interleukin-10 (IL-10) [51]. Such alterations in the cytokine profile can favor pro-inflammatory responses.

The effects of NNK and nicotine in pancreatitis appear to be very complex and somewhat paradoxical. The findings from these separate studies though are not as irreconcilable as they first appear. Early pancreatitis events likely involve a direct interaction of NNK/nicotine with α7 nAChR localized on acini, competing with a potential anti-inflammatory phase via α7 nAChR on macrophages [39,47,48,49]. It is possible that anti-inflammatory modulation of macrophages induces the healing process (perhaps through TGF-β1 signaling). In rodents pancreatic damage is normally resolved within a few days and cigarette smoke-induced immune modulation may turn this into an ongoing process.

The anti-inflammatory response could be short-lived, however, as sustained contact with cigarette toxins eventually gives way to chronic pancreatic inflammation [52]. These chronic inflammatory responses potentially occur through uptake and metabolism (bioactivation) of NNK/nicotine in macrophages. Bioactivation of cigarette toxins in the pancreas itself may contribute to pancreatic cancer [53], but it has not been determined if this process influences smoking-related pancreatitis. Limited evidence suggests bioactivation of NNK or nicotine in acini might contribute to pathological responses which predispose to pancreatitis. This is discussed further in Section 3.4.

### 3.4. “Bioactivation” of Toxins in the Pancreas

P450 enzymes, critical for bioactivation of NNK, have been identified in rodent pancreas (isoforms 2B6, 3A5 and 2A3), although there have been variable results in human pancreas [54]. There was no evidence of P450 enzymes in human pancreatic samples from smokers and non-smokers in one study which employed cytochemical detection methods [55]. However, another report identified CYP450 enzymes in human pancreatic tissue using immunohistochemical techniques [56]. Moreover, the levels of enzymes were elevated in samples from patients with CP and pancreatic cancer [56].

More recent findings have demonstrated that NNK may induce changes at the genetic level within the pancreatic acinar cell itself [57]. Whether this occurs via a “bioactivated” form of NNK or an alternative pathway remains uncertain. The vitamin thiamin (vitamin B1) is crucial for pancreatic function due to its involvement in oxidative energy metabolism and its role as a cofactor for multiple enzymes in mitochondrial ATP production [58]. The pancreatic acinar cell maintains high thiamin levels through uptake from its environment via thiamin transporters-1 and -2 (THTR-1 and THTR-2). Protein and mRNA levels of these transporters decreased significantly when pancreatic acinar 266-6 cells were exposed to NNK. These changes were linked with a reduction in thiamin uptake and thiamin transporter promoters- SLC19A2 and SLC19A3. Extended periods of NNK treatment in mice gave comparable results [57]. This study emphasizes how a cigarette toxin such as NNK can induce modifications in pancreatic cell function at the level of transcription, giving rise to, in this circumstance, thiamin deficiency. Low intracellular levels of thiamin would ultimately impair oxidative energy metabolism, increase oxidative stress and compromise the structure and function of mitochondria [59]. The resulting drop in cellular ATP levels, might play a role in sensitizing the pancreas to a secondary insult, predisposing it to development of pancreatitis [60].

### 3.5. β-Adrenergic Receptors

NNK structurally resembles classic β-adrenergic agonists and has high affinity for human β-1 and β-2 receptors (EC_50_ for β1= 5.8 nM; EC_50_ for β2 = 128 nM) [61]. In mammalian cells, stimulation of β-adrenergic receptors activates the enzyme adenylate cyclase resulting in elevations of the intracellular second messenger cAMP. Moreover, cAMP has been shown to participate in pancreatitis responses such as zymogen activation and amylase secretion [62]. A study by Alexandre *et al.* discovered β-adrenergic receptors in rat pancreatic acinar cells, although NNK mediated zymogen activation was not reduced when β-adrenergic receptors were blocked with the inhibitor propranolol [63]. Stimulation of β-adrenergic receptors can also cause release of arachidonic acid and it is possible that NNK could potentiate such release through the enzyme phospholipase A2 (PLA2), an important component in inflammation. Various PLA2 isoforms, namely phospholipase A2-II and A2-IV, are elevated during human AP and may influence disease severity both locally and systemically [64]. Whether NNK mediates arachidonic acid release, however, and additional pancreatitis responses through β-adrenergic receptors remains to be seen.

## 4. Conclusions

The amount of knowledge regarding the pathogenesis of smoking-induced pancreatitis is scarce compared with what is known about other etiologies of the disease, such as alcohol abuse and gallstones. Improvement and expansion of trustworthy animal models of smoking-related pancreatitis, as well as human studies, are needed if relevant cellular targets and effective therapies are to be identified.

**Figure 1:**
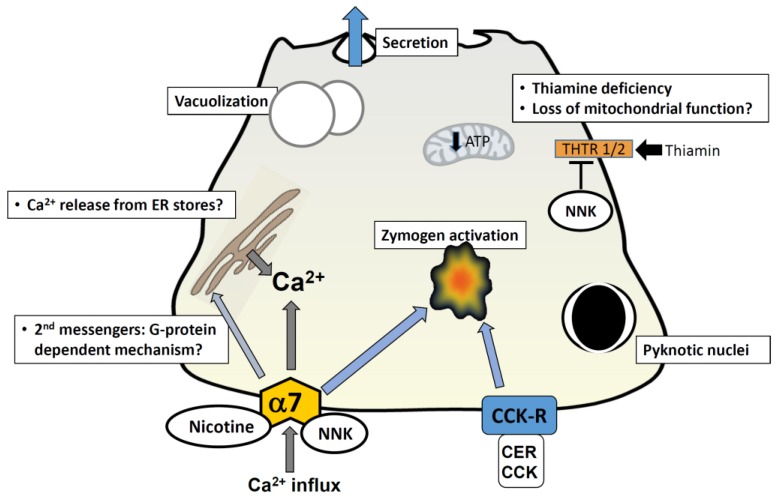
Cellular mechanisms mediated by cigarette toxins in pancreatic acinar cells. A representation of a pancreatic acinar cell is shown. Exposure to nicotine and NNK is known to cause morphological changes comparable to those seen in pancreatitis; these include vacuolization and formation of pyknotic nuclei. Nicotine has been shown to stimulate secretion by itself and augment cholecystokinin-mediated (CCK) secretion at low concentrations (100 µM); at higher concentrations (>1 mM) it inhibits secretion. Nicotine-mediated events are abrogated by pre-treatment of cells with the alpha-7 nAChR blocker mecamylamine, and the calcium channel antagonist 2-APB; this implies that nicotine-induced events occur via the alpha-7 nAChR and an elevation in intracellular calcium. This may be via a direct influx of calcium through the nAChR (given that it is a calcium channel) or through a G-protein coupled mechanism which gives rise to second messengers and subsequent release from intracellular calcium stores in the ER. NNK induces zymogen activation in acini and augments cerulein (CER)-induced zymogen activation; this effect is abrogated by the nAChR blocker mecamylamine and in alpha-7^−/−^ mice. NNK has been shown to inhibit uptake of the vitamin thiamin, by reducing levels of thiamin transporters; thiamin is crucial for pancreatic function due to its involvement in oxidative energy metabolism and its role as a cofactor for multiple enzymes in mitochondrial ATP production. Thiamin deficiency ultimately impairs oxidative energy metabolism, increases oxidative stress and compromises the structure and function of mitochondria. The resulting drop in cellular ATP levels, might play a role in sensitizing the pancreas to a secondary insult, predisposing it to development of pancreatitis. Abbreviations: CER = cerulein; CCK = cholecystokinin; CCK-R = CCK receptor; THTR = thiamin transporters; α7 and α7nAChR = α7 nicotinic acetylcholine receptor.

Some models described in this review, which focus on specific cigarette toxins, such as nicotine and it’s more potent derivative NNK, have yielded encouraging findings (summarized in Figure 1 below). For instance, an α7nAChR in pancreatic acinar cells has emerged as a likely therapeutic target, given its role in premature zymogen activation and related pancreatitis responses [63]. This receptor, along with β-adrenergic receptors, also mediates NNK activation of cellular signals including the enzyme cyclooxygenase-2 (COX2), epidermal growth factor receptor (EGFR) and extracellular-signal-regulated kinases (ERK) in pancreatic cancer cells and ductal cells [65,66]. Furthermore, pancreatic stellate cells have been shown to express α7nAChR and respond upon exposure to nicotine, resulting in increased proliferation and extracellular matrix (ECM) production [25]. ECM production has been shown to contribute to the survival of pancreatic cancer cells and resistance to apoptosis [67]. In addition, NNK has been shown to effect changes at the genetic level which could sensitize to pancreatitis and also cause pancreatic cancer. For instance, NNK triggers thiamin deficiency in acinar cells (a condition which could leave the pancreas vulnerable to pancreatitis) by affecting transcription of thiamin transporters [57], whereas it predisposes to cancer through formation of DNA adducts and genetic mutations [53]. Thus, cellular targets of cigarette toxins and downstream pathways involved in pancreatitis, may also have relevance in progression to pancreatic cancer.

Finally, factors such as epigenetic changes or environmental stimuli, may further propagate pancreatic injury when combined with smoking. Studies looking at smoking by itself, and with other risk factors, will greatly improve understanding of mechanisms underlying smoking-induced pancreatic diseases.
